# Health status correlates of malnutrition diagnosed based on the GLIM criteria in older Polish adults—Results of the PolSenior 2 study

**DOI:** 10.1371/journal.pone.0317011

**Published:** 2025-01-07

**Authors:** Aleksandra Kaluźniak-Szymanowska, Ewa Deskur-Śmielecka, Roma Krzymińska-Siemaszko, Arkadiusz Styszyński, Sławomir Tobis, Marta Lewandowicz, Jerzy Chudek, Tomasz Kostka, Małgorzata Mossakowska, Karolina Piotrowicz, Hanna Kujawska-Danecka, Katarzyna Wieczorowska-Tobis

**Affiliations:** 1 Department of Palliative Medicine, Poznan University of Medical Sciences, Poznan, Poland; 2 Department of Occupational Therapy, Poznan University of Medical Sciences, Poznan, Poland; 3 Department of Internal Diseases and Oncological Chemotherapy, Faculty of Medical Sciences in Katowice, Medical University of Silesia, Katowice, Poland; 4 Department of Geriatrics Medical University of Lodz Healthy Ageing Research Centre (HARC), Lodz, Poland; 5 Study on Aging and Longevity, International Institute of Molecular and Cell Biology, Warsaw, Poland; 6 Department of Internal Medicine and Gerontology, Faculty of Medicine, Jagiellonian University Medical College, Krakow, Poland; 7 Department of Rheumatology, Clinical Immunology, Geriatrics and Internal Medicine, Medical Univeristy of Gdansk, Gdansk, Poland; Instituto Nacional de Geriatria, MEXICO

## Abstract

**Introduction:**

Older individuals are at risk of malnutrition resulting from chronic diseases-related body and muscle mass reduction. In turn, nutritional deficiencies may enhance catabolic processes, leading to accelerated aging and comorbidity, thus creating a vicious cycle. Our study aimed to assess the prevalence of malnutrition using the Global Leadership Initiative on Malnutrition (GLIM) criteria and to determine the health correlates of malnutrition in a representative sample of community-dwelling older adults.

**Methods:**

We used the GLIM criteria to diagnose malnutrition in 5,614 participants of the PolSenior2 study. The PolSenior2 study was a population-based survey designed to assess the medical, psychological, social, and economic characteristics of community-dwelling older adults.

**Results:**

Malnutrition was diagnosed in 13.4% of the participants using the GLIM criteria. Results of multiple logistic regression showed that the risk of depression [OR 4.18, p<0.001], peptic ulcer disease [OR 2.73, p<0.001], past stroke [OR 1.71, p<0.001], cognitive impairment [OR 1.34, p = 0.015], and chronic pain [OR 1.23, p = 0.046] were independent correlates of malnutrition.

**Conclusion:**

Due to the high risk of malnutrition, special attention should be paid to individuals in late old age. Suspected malnutrition should also be considered in people at risk of depression, with peptic ulcer disease, past stroke, and cognitive impairment. Chronic pain should also prompt the diagnosis for malnutrition.

## Introduction

Older adults are the most rapidly growing age group in the world. Therefore, maintaining good health conditions, functional fitness, and high quality of life are priorities in the care of the older population [1, 2]. Advanced age is associated with a higher risk of chronic diseases, which may diminish body and muscle mass and increase the risk of malnutrition [2, 3]. Nutritional deficiencies, in turn, may activate catabolic processes leading to accelerated aging and comorbidity occurrence, thus creating a vicious cycle [3–5]. Therefore, maintaining good nutritional status is a crucial determinant of quality of life and functional capacity in later years.

Older adults are particularly vulnerable to malnutrition, originating from aged-associated changes, socioeconomic factors, and overall poor health conditions [1, 3, 5, 6]. Based on the results of population studies (PolSenior and PolSenior2), we have previously described socioeconomic correlates of malnutrition (female sex, older age, unmarried status in men, subjective loneliness, and self-reported poverty), emphasizing that some are modifiable [7, 8]. However, most cases of malnutrition in developed countries derive from disease conditions (both chronic and acute) [2, 3]. Thus, determining health correlates will enable the identification of subjects at risk of malnutrition and the early implementation of preventive strategies.

Our previous analysis of the PolSenior population showed that depressive symptoms, cognitive impairment, multimorbidity, anemia, and total edentulism were independent risk factors for poor nutritional status. That analysis assessed nutritional status solely with the Mini Nutritional Assessment- Short Form (MNA-SF) questionnaire [9]. Meanwhile, in 2019, the Global Leadership Initiative on Malnutrition (GLIM) experts proposed new diagnostic criteria for malnutrition. According to these criteria, malnutrition can be diagnosed if the results of screening tools, such as MNA-SF, are confirmed with phenotypic and etiologic aspects [10]. Despite these recommendations, the assessment of nutritional status is often neglected in everyday clinical practice, particularly in non-institutional settings. To our knowledge, population-based studies using the new malnutrition criteria have yet to be performed.

Our study aimed to assess the prevalence of malnutrition using the GLIM criteria which are the newest and currently recommended, and determine its health correlates in a representative sample of community-dwelling older adults in Poland.

## Material and methods

The PolSenior2 study was conducted from 01 September 2018 to 20 December 2019. It assessed the medical, psychological, social, and economic characteristics of community-dwelling older adults in Poland. A representative sample of 5987 persons aged 60 or more was enrolled. Participants were selected by a multi-step procedure, independently for each of the seven 5-year age groups. The detailed sampling procedure and the construction of the study questionnaires have been described previously [11]. The Bioethical Committee at the Medical University of Gdańsk approved the study protocol (NKBBN/257/2017). Respondents or caregivers gave written informed consent to participate in the project.

### 1. Nutritional status assessment

**Fig 1:**
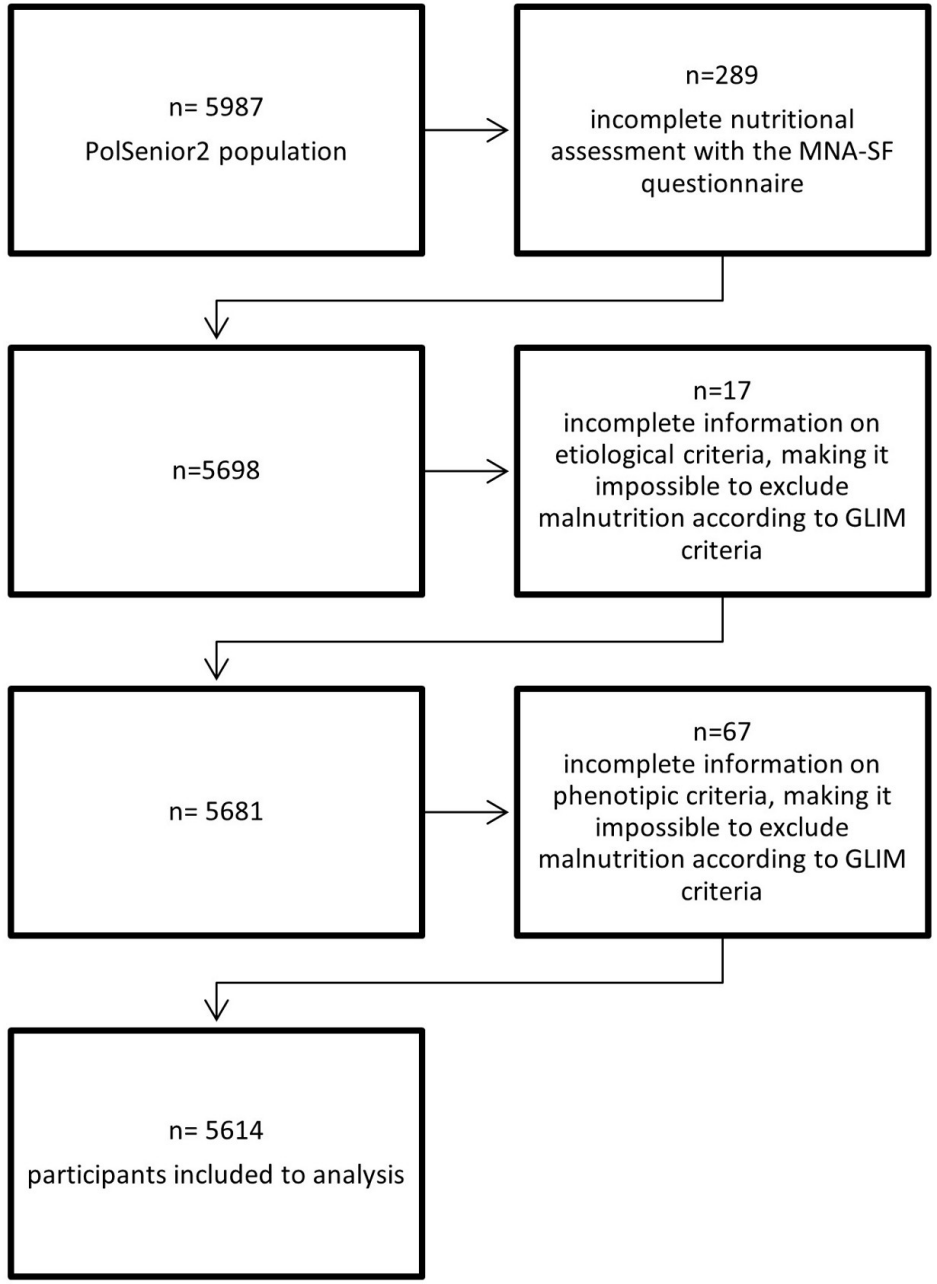
The process of selection of participants.

Five-thousand-six-hundred-fourteen respondents (2864 women and 2750 men) were assessed for malnutrition using the GLIM criteria. Data concerning the nutritional status of the remaining 373 participants (6.2% of the PolSenior2 cohort) were incomplete and excluded from analysis (Fig 1).

In the screening stage, the MNA-SF questionnaire was used [12, 13]. The maximum possible score is 14 points. Further assessment for malnutrition was conducted in those who scored 11 points or less.

Following the recommendations of GLIM experts, phenotypic and etiological criteria were used to diagnose malnutrition. Participants fulfilling at least one criterion from each category were diagnosed as malnourished (Table 1).

**Table 1:** GLIM phenotypic and etiological criteria.

Phenotypic criteria:	Etiological criteria:
Weight loss—unintentional loss of >5% of body weight in the last six months (based on respondents’ answers).	Respondents reporting any decline in food intake in the last three months in the MNA-SF questionnaire were recognized as having reduced food intake.
Low body mass index (BMI)–<20 kg/m^2^ in people up to 70 years of age and <22 kg/m^2^ in those aged 70 and over.	The presence of disease associated with chronic or recurrent inflammation was determined based on interviews with the respondents. Additionally, we measured the C-reactive protein (CRP) levels in all participants to assess inflammation during the survey. The CRP ≥5 mg/L fulfilled the etiological criterion.
Low muscle mass (LMM)–assessed based on calf circumference measurement. Low muscle mass was diagnosed if the calf circumference was ≤33 cm in women and ≤34 cm in men [14–16].	

### 2. Neuropsychological assessment

The cognitive functions were assessed using the Mini-Mental State Examination (MMSE) tool corrected for age and education [17]. The normal test result ranged from 27 to 30 points. Possible cognitive impairment was classified as follows:

> 27–24 pts: mild cognitive impairment
> 24–19 pts: mild dementia
> 19–11 pts: moderate dementia
> 11–0 pts: severe dementia

The risk of depression was assessed using the 15-item Geriatric Depression Scale (GDS) [18]. To include people with a very low level of education and those with vision problems, the interviewers filled in the questionnaire based on verbal answers provided by the participants (in its original version, examined persons should complete the GDS questionnaire without any help). To exclude unreliable answers, people whose MMSE results indicated moderate or severe dementia did not fill out the GDS questionnaire. Depression risk was considered in participants with a GDS score of 6 points or more.

### 3. Medical interview

The PolSenior2 study interviewers asked respondents or their caregivers about selected diseases potentially influencing their nutritional status (peptic ulcer disease, past stroke, Parkinson’s disease, cancer currently or in the past, chronic obstructive pulmonary disease, diabetes, hypertension, liver cirrhosis). They also collected information about participants’ medication. The analysis included prescription and over-the-counter drugs taken at least once a week. Based on the number of medications taken, the respondents were divided into three groups: without polypharmacy (up to 4 drugs taken), with polypharmacy (5–9 medications), and with excessive polypharmacy (at least ten drugs taken). The occurrence of chronic pain, defined as pain lasting longer than three months, was assessed. The respondents were classified with edentulism based on information about their dental status (number of teeth). Interviewers also asked about falls in the last 12 months.

### 4. Statistical analysis

Statistical analysis was performed using the STATISTICA 10 PL software package (Stat Soft, Poland). The relationships between categorical variables were examined with Pearson’s chi-square test. Logistic regression analysis (univariate and multivariate) was conducted to determine which variables correlate the occurrence of malnutrition based on the GLIM criteria. The odds ratio and confidence interval were chosen, with a confidence limit of 95%. A p-value of less than 0.05 was considered significant.

The study sample included an equal number of men and women in all age cohorts, making older groups and men overrepresented compared to the actual population structure. Post-stratification was necessary to make the sample representative of the Polish population.

## Results

### 1. Characteristics of persons at risk of malnutrition

The mean age of participants was 74.7 ± 9.4 years, and women constituted 51.0% of the study sample. A positive malnutrition screening test result (MNA-SF 11 points or less) was found in 27.8% of subjects. Importantly, women were significantly more likely to obtain a positive screening test result compared to men (30.9% vs. 24.8%, p<0.001). Based on the GLIM criteria, malnutrition was diagnosed in 13.4% of the participants. Including post-stratification, the prevalence of malnutrition was estimated at 11.2% (CI 9.9–12.5) in women and 10.6% (CI 8.8–12.3) in men. Sex did not influence the prevalence of abnormal nutritional status.

Table 2 shows the frequency of specific phenotypic and etiologic criteria for malnutrition in participants with a positive screening test result (MNA-SF positive). At least one phenotypic criterion was fulfilled in 67.6% of respondents at risk of malnutrition, and at least one etiologic criterion was found in 69.6% of individuals at risk. The frequency of unintentional weight loss (p = 0.022), disease associated with chronic or recurrent inflammation (p<0.001), and the presence of at least one phenotypic criterion (p = 0.005) was higher in men than in women.

**Table 2:** Frequency of phenotypic and etiologic GLIM criteria in individuals with a positive screening test for malnutrition (MNA-SF positive).

Variable			Sex		p-value
		Respondents MNA-positive n (%)	Women n (%)	Men n (%)	
**Unintentional weight loss**	No	893 (100.0)	527 (59.0)	366 (41.0)	0.022
	Yes	669 (100.0)	356 (53.2)	313 (46.8)	
**Body mass index**	Normal	1260 (100.0)	705 (56.0)	555 (44.0)	0.639
	Low	266 (100.0)	153 (57.5)	113 (42.5)	
**Calf circumference (low muscle mass)**	Normal	1024 (100.0)	585 (57.1)	439 (42.9)	0.530
	Low	514 (100.0)	285 (55.4)	229 (44.6)	
**At least one phenotypic criterion**	No	508 (100.0)	313 (61.6)	195 (38.4)	0.005
	Yes	1060 (100.0)	573 (54.1)	487 (45.9)	
**Reduced food intake**	No	943 (100.0)	515 (54.6)	428 (45.4)	0.063
	Yes	625 (100.0)	371 (59.4)	254 (40.6)	
**Inflammation**	No	1131 (100.0)	653 (57.7)	478 (42.3)	0.113
	Yes	437 (100.0)	233 (53.3)	204 (46.7)	
**Disease associated with chronic or recurrent inflammation**	No	1054 (100.0)	628 (59.6)	426 (40.4)	<0.001
	Yes	514 (100.0)	258 (50.2)	256 (49.8)	
**At least one etiological criterion**	No	477 (100.0)	278 (58.3)	199 (41.7)	0.348
	Yes	1091 (100.0)	608 (55.7)	483 (44.3)	

### 2. Health factors associated with malnutrition diagnosed by GLIM criteria—Univariate analysis

**Fig 2:**
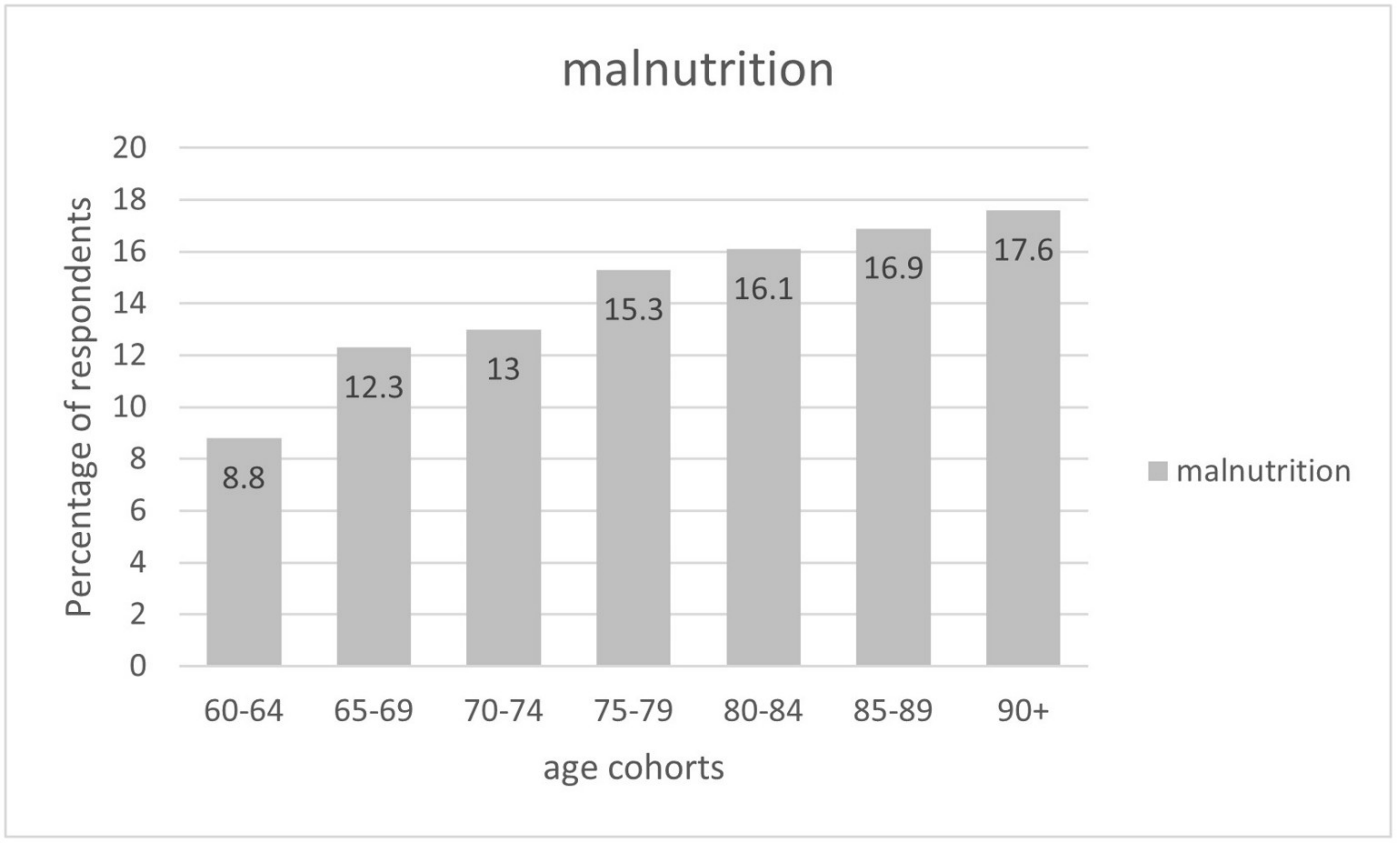
The frequency of malnutrition in the Polish older adults stratified by age.

Tables 3 and 4 show the correlates of health factors, age, and sex on the frequency of malnutrition diagnosed based on the GLIM criteria. Age significantly affected the frequency of malnutrition, with individuals over 80 years being at the highest risk (p<0.001) (Fig 2).

**Table 3:** Correlates of health factors, age, and sex on the prevalence of malnutrition diagnosed with the GLIM criteria.

Variable		Malnutrition (GLIM)		p-value
		No	Yes	
		n (%)	n (%)	
**Sex**	Women	2468 (50.8)	396 (52.7)	0.312
	Men	2395 (49.2)	355 (47.3)	
**Age (years)**	60–64	828 (17.0)	66 (8.8)	<0.001
	65–69	974 (20.0)	92 (12.3)	
	70–74	912 (18.8)	98 (13.0)	
	75–79	740 (15.2)	115 (15.3)	
	80–84	665 (13.7)	121 (16.1)	
	85–89	422 (8.7)	127 (16.9)	
	≥ 90	322 (6.6)	132 (17.6)	
**Number of drugs**	0–4	2409 (49.5)	242 (32.2)	<0.001
	5–9	1990 (40.9)	389 (51.8)	
	≥ 10	464 (9.5)	120 (16.0)	
**Parkinson’s disease**	No	4792 (98.5)	732 (97.5)	0.030
	Yes	71 (1.5)	19 (2.5)	
**Depression risk**	no	3553 (78.1)	232 (40.7)	<0.001
	Yes	996 (21.9)	338 (59.3)	
**Cognitive impairment**	No	3816 (78.5)	396 (52.7)	<0.001
	Yes	1047 (21.5)	355 (47.3)	
**Peptic ulcer disease**	No	4275 (87.9)	556 (74.0)	<0.001
	Yes	588 (12.1)	195 (26.0)	
**Liver cirrhosis**	No	4828 (99.3)	744 (99.1)	0.530
	Yes	35 (0.7)	7 (0.9)	
**Cancer (currently or in the past)**	No	4405 (90.6)	665 (88.5)	0.080
	Yes	458 (9.4)	86 (11.5)	
**Past stroke**	No	4499 (92.5)	619 (82.4)	<0.001
	Yes	364 (7.5)	132 (17.6)	
**Chronic pain**	No	2616 (53.8)	308 (41.0)	<0.001
	Yes	2247 (46.2)	443 (59.0)	
**Falls**	No	4043 (83.1)	513 (68.3)	<0.001
	Yes	820 (16.9)	238 (31.7)	
**Diabetes**	Normal	2569 (52.8)	390 (51.9)	0.002
	pre-diabetes	1080 (22.2)	135 (18.0)	
	Diabetes	1214 (25.0)	226 (30.1)	
**Hypertension**	No	1115 (22.9)	168 (22.4)	0.735
	Yes	3748 (77.1)	583 (77.6)	
**Anaemia**	No	3980 (86.7)	610 (85.8)	0.505
	Yes	610 (13.3)	101 (14.2)	
Edentulism	No	2950 (60.7)	338 (45.0)	<0.001
	Yes	1913 (39.3)	413 (55.0)	

**Table 4:** Factors associated with malnutrition (univariate analysis).

	Total		Women		Men	
	OR (95%Cl)	p-value	OR (95%Cl)	p-value	OR (95%Cl)	p-value
**Age (years)**	1.04 (1.03–1.05)	<0.001	1.05 (1.04–1.07)	<0.001	1.03 (1.02–1.05)	<0.001
**Number of drugs: 5–9**	1.82 (1.48–2.24)	<0.001	2.17 (1.62–2.89)	<0.001	1.52 (1.14–2.04)	0.005
**Number of drugs ≥10**	2.57 (1.93–3.42)	<0.001	2.57 (1.72–3.85)	<0.001	2.59 (1.73–3.89)	<0.001
**Parkinson’s disease**	2.10 (1.16–3.81)	0.014	1.75 (0.72–4.29)	0.218	2.47 (1.11–5.51)	0.027
**Depression risk**	5.34 (4.40–6.48)	<0.001	5.00 (3.81–6.55)	<0.001	5.83 (4.41–7.71)	<0.001
**Cognitive impairment**	2.22 (1.81–2.74)	<0.001	2.32 (1.74–3.10)	<0.001	2.12 (1.57–2.87)	<0.001
**Peptic ulcer disease**	2.73 (2.20–3.39)	<0.001	3.42 (2.54–4.62)	<0.001	2.17 (1.59–2.97)	<0.001
**Liver cirrhosis**	1.25 (0.49–3.23)	0.643	2.14 (0.59–7.73)	0.244	0.78 (0.18–3.34)	0.735
**Cancer (current on in the past)**	1.26 (0.94–1.68)	0.121	1.19 (0.79–1.80)	0.413	1.33 (0.89–1.99)	0.168
**Past stroke**	2.49 (1.89–3.28)	<0.001	2.36 (1.55–3.60)	<0.001	2.63 (1.83–3.80)	<0.001
**Chronic pain**	1.84 (1.52–2.22)	<0.001	1.94 (1.48–2.55)	<0.001	1.75 (1.34–2.29)	<0.001
**Falls**	1.99 (1.61–2.46)	<0.001	1.82 (1.37–2.42)	<0.001	2.25 (1.63–3.12)	<0.001
**Pre-diabetes**	0.88 (0.69–1.13)	0.319	1.08 (0.77–1.52)	0.656	0.72 (0.50–1.04)	0.081
**Diabetes**	1.38 (1.11–1.70)	0.003	1.36 (1.00–1.85)	0.048	1.39 (1.03–1.87)	0.033
**Hypertension**	1.13 (0.90–1.41)	0.294	1.15 (0.84–1.57)	0.393	1.11 (0.80–1.53)	0.532
**Aneamia**	1.09 (0.83–1.43)	0.516	0.96 (0.65–1.42)	0.822	1.25 (0.86–1.82)	0.244
Edentulism	1.56 (1.30–1.88)	<0.001	1.58 (1.22–2.05)	<0.001	1.54 (1.18–2.01)	0.002

Malnutrition was also more prevalent in individuals taking more than four drugs compared to those without polypharmacy (p<0.001). Considering the health factors, malnutrition was diagnosed more often in people with depression risk, cognitive impairment, Parkinson’s disease, chronic pain, history of falls in the past 12 months, past stroke, peptic ulcer disease, diabetes, and in those with edentulism.

### 3. Results of the multivariate regression analysis

Table 5 presents multiple regression models, including all correlates of malnutrition in the univariate analysis. The strongest correlates of malnutrition were depression risk (OR 4.18) and age over 90 (OR 3.7). Other independent risk factors contributing to malnutrition were peptic ulcer disease, past stroke, chronic pain and cognitive impairment.

**Table 5:** Factors associated with malnutrition (multiple logistic regression). Only complete data were included for multivariate analysis.

	Total		Women		Men	
	n = 5614		n = 2864		n = 2750	
	OR (95%Cl)	p-value	OR (95%Cl)	p-value	OR (95%Cl)	p-value
**Age 60–64 (Ref.)**						
**Age: 65–69 years**	1.10 (0.78–1.55)	0.580	1.28 (0.79–2.07)	0.320	0.97 (0.9–1.57)	0.888
**Age: 70–74 years**	1.13 (0.80–1.59)	0.480	1.07 (0.65–1.77)	0.787	1.19 (0.74–1.90)	0.479
**Age: 75–79 years**	1.53 (1.09–2.15)	0.014	2.06 (1.28–3.31)	0.003	1.11 (0.68–1.82)	0.683
**Age: 80–84 years**	1.55 (1.10–2.18)	0.012	1.98 (1.22–3.20)	0.005	1.18 (0.72–1.94)	0.503
**Age: 85–89 years**	2.55 (1.80–3.63)	<0.001	3.31 (2.01–5.44)	<0.001	2.02 (1.22–3.35)	0.006
**Age: ≥ 90 years**	3.70 (2.59–5.30)	<0.001	5.28 (3.16–8.85)	<0.001	2.62 (1.58–4.34)	<0.001
**Numer of drugs 0–4 (Ref.)**						
**Number of drugs: 5–9**	1.19 (0.94–1.49)	0.146	1.33 (0.97–1.83)	0.081	1.08 (0.77–1.50)	0.662
**Number of drugs: ≥ 10**	1.21 (0.87–1.69)	0.267	1.19 (0.76–1.87)	0.441	1.28 (0.79–2.08)	0.318
**Parkinson’s disease**	1.12 (0.59–2.12)	0.738	-	-	1.44 (0.59–3.54)	0.421
**Depression risk**	4.18 (3.39–5.16)	<0.001	3.91 (2.91–5.25)	<0.001	4.56 (3.37–6.16)	<0.001
**Cognitive impairment**	1.34 (1.06–1.71)	0.015	1.37 (0.98–1.92)	0.069	1.28 (0.91–1.81)	0.160
**Peptic ulcer disease**	2.73 (2.16–3.45)	<0.001	3.67 (2.64–5.10)	<0.001	2.02 (1.44–2.84)	<0.001
**Past stroke**	1.71 (1.27–2.32)	<0.001	1.75 (1.10–2.77)	0.018	1.67 (1.11–2.51)	0.014
**Chronic pain**	1.23 (1.00–1.52)	0.046	1.24 (0.92–1.67)	0.153	1.22 (0.91–1.63)	0.191
**Falls**	1.15 (0.90–1.45)	0.261	1.08 (0.79–1.49)	0.615	1.22 (0.84–1.77)	0.299
**Diabetes: pre-diabetes**	0.95 (0.73–1.24)	0.723	-	-	0.83 (0.56–1.22)	0.338
**Diabetes: diabetes**	1.21 (0.96–1.54)	0.112	-	-	1.26 (0.90–1.77)	0.183
Edentulism	1.19 (0.97–1.46)	0.105	1.13 (0.84–1.51)	0.412	1.28 (0.95–1.72)	0.105

No differences were found in multiple regression models analyzed separately for women and men (Table 5).

## Discussion

To the best of our knowledge, this is the first population-based study to determine the health correlates of malnutrition diagnosed using the GLIM criteria. Our results indicate that more than one in every seven older individuals in Poland is malnourished. This percentage is lower compared to our previous analysis, in which nearly every second participant had poor nutritional status. This discrepancy can be attributed to the fact that the previous diagnosis of malnutrition was based exclusively on the MNA-SF score [9], which is only a screening tool within the GLIM criteria. Comparable findings were reported by Rodriguez-Sanchez et al. [19], who analyzed data from 1,660 older people (mean age 75.6 years) in Toledo, Spain, and found that 12.6% (n = 209) were malnourished based on the GLIM criteria. However, one study shows a higher percentage of malnutrition. For instance, Demirdag et al. [20] found that 24.5% (n = 139) of 569 older individuals (mean age 74.4 years) in Turkey were malnourished according to the GLIM criteria. Nevertheless, further research in this area is needed, preferably population-based studies.

Similarly to our previous analysis of the correlates of malnutrition diagnosed using the MNA-SF score [9], we observed the highest risk of poor nutritional status in individuals in their late old age. These findings align with the results of a systematic review by Bardon et al. [21], which analyzed the most common socioeconomic risk factors and determinants of malnutrition. There are several reasons for poorer nutritional status in older adults, including: gerostomatological problems that make it difficult to consume certain groups of products like meat, changes in the digestive tract, such as intestinal peristalsis disorders that are associated with discomfort and stomach pain and therefore aversion to eat, faster feeling of satiety or economic problems [3, 21]. However, our study revealed a gender difference: the relationship was significant for women after the age of 75, while for men, it became significant at age 85. The PolSenior study indicates that older women in Poland, as in many other countries, are much more likely to experience loneliness than men [22, 23], which may contribute to this gender difference.

Notably, our analysis did not show a significant difference in the incidence of malnutrition between sexes, regardless of age. This contrasts with the previous results of the PolSenior study (2008–2011), where malnutrition was diagnosed significantly more often in women [9]. However, it should be noted that the previous analysis relied solely on the MNA-SF questionnaire to diagnose poor nutritional status. In the current analysis, women were also more likely to obtain a positive screening test result (MNA-SF). However, in further diagnostics, men were more likely to be diagnosed with at least one phenotypic criterion and a disease associated with chronic or recurrent inflammation, which constitutes the etiological criterion of malnutrition.

We identified the risk of depression as the strongest health correlate of malnutrition [OR 4.18 (3.39–5.16)]. Depression as a risk factor for malnutrition has been investigated by numerous scientists [24–26]. Its presence is associated with lower motivation to engage in various activities, including those related to preparing and eating meals. For example, Vandewoude et al. [27] reported that depression was more than twice as common in community-dwelling individuals with malnutrition diagnosed using the MNA-SF questionnaire compared to adequately nourished individuals (68% vs. 32%). Conversely, Torres et al. [28] observed a significantly higher odds ratio for malnutrition in individuals with depression living in urban areas, but not in rural areas [OR 20.67 (95% CI: 17.46–24.49)]. Similarly, Wei et al. [29] in their analysis of 4,916 people over 60 years old, found the odds ratio for malnutrition in individuals with depression to be 1.31. Such diverse results may stem from the use of different diagnostic criteria for depression and the selection of study populations.

Peptic ulcer disease was also a strong health correlate of malnutrition in our analysis [OR 2.73 (2.16–3.45)]. Similar results were found by Kiesswetter et al. [30], who assessed the health correlates of malnutrition in older people from various environments and noticed that gastrointestinal diseases were the strongest correlates in community-dwelling older people. We also observed an association between past stroke and malnutrition [OR 1.71 (1.27–2.32)]. Strokes frequently lead to functional disabilities, preventing older individuals from preparing nutritious meals. The consequences of a stroke may also include intensified catabolic processes, polypharmacy, cognitive impairment, increased risk of falls, and dysphagia [31]. All these effects can directly or indirectly increase the risk of malnutrition among older adults. More than one out of four stroke survivors in our sample (26.6%) were diagnosed with malnutrition according to the GLIM criteria. This ratio is similar to the results obtained by Nozoe et al. [32], who used the same criteria and found malnutrition in 28.7% of patients with a history of stroke. The prevalence of malnutrition in such subjects in other studies varied from 6.1% to 62%, depending on the diagnostic criteria of malnutrition, the time elapsed since the stroke, and its severity [33].

We identified chronic pain as a health problem correlating with malnutrition in older individuals [OR 1.33 (1.12–1.58)]. Similarly, Bauer et al. [34], in their study involving 3,406 hospitalized older people (mean age 77 years), reported a significantly higher risk of malnutrition in participants with pain compared to those not reporting pain [OR 1.44 (1.17–1.77)]. Moreover, the meta-analysis by Stubbs et al. [35] demonstrated an increased risk of falls (up to 80%) in subjects with chronic pain [OR 1.80 (1.56–2.09)].

Another health correlate of malnutrition identified in our study was cognitive impairment. A recently published meta-analysis by Arifin et al. [36], which included 16 studies involving 6,513 older individuals living in various settings, showed that malnutrition was present in 32.5% of people with dementia, while 46.8% were at risk of malnutrition. The authors indicated that the causes might include behavioral disorders, appetite disorders, apathy, or depression.

Although we cannot determine the direction of the cause-effect relationship between malnutrition and health factors, individuals with at least one identified health correlate should be considered at risk of malnutrition and should undergo regular nutritional assessments.

Our work has certain limitations. We assessed muscle mass as a phenotypic criterion of malnutrition based on calf circumference measurement instead of using DEXA (Dual-energy X-ray absorptiometry) or BIA (Bioelectrical Impedance Analysis) which are more accurate mhetods, but in everyday practice, using a tape measure is cheaper, easier, more common (due to the lack of contraindications) and allows for easy repetition at subsequent visits. In addition, the risk of depression and cognitive impairment was assessed using screening tools only and was not verified by specialists. Furthermore, the size of the subgroup with malnutrition may be insufficient to demonstrate significance in stratified analyses (e.g., gender subgroups for pain and cognitive impairment).

However, the presented analysis also has several strengths. Firstly, it is a large population-based study, allowing the conclusions to be extrapolated to the entire population of community-dwelling older adults in Poland and potentially other well-developed countries. Secondly, this is the first study investigating malnutrition in a European population using the GLIM diagnostic criteria. It should be emphasized that applying the GLIM criteria allowed us to diagnose and analyze actual malnutrition, not just its risk.

## Conclusion

In conclusion, women were more likely to have a positive screening test result, while men were more likely to be diagnosed with unintentional weight loss and diseases associated with chronic or recurrent inflammation. Due to the high risk of malnutrition, special attention should be paid to individuals with depression and advanced age. It should be emphasized that women over the age of 75 are particularly vulnerable to malnutrition, while men over the age of 85 are also at significant risk. Malnutrition should also be suspected in people with peptic ulcer disease, post-stroke conditions, and cognitive impairment. Additionally, chronic pain should prompt a diagnosis for malnutrition.
